# New recipes for old ingredients: high doses of methylprednisolone and verapamil in cluster headache

**DOI:** 10.1186/1129-2377-14-S1-P59

**Published:** 2013-02-21

**Authors:** G Zanchin, C Disco, F Mainardi, E Mampreso, C Lisotto, F Maggioni

**Affiliations:** 1 Headache Centre of Veneto Region, Department of Neurosciences, University of Padua, Italy; 2 Headache Centre of the Veneto Region, Department of Neurosciences, University of Padua, Italy; 3 Headache Centre of SS Giovanni e Paolo Hospital, Venice, Italy; 4 Headache Centre of Piove di Sacco Hospital (Padua), Italy; 5 Headache Centre of of S. Vito al Tagliamento Hospital, Italy

## 

Corticosteroids (C) rapidly suppress Cluster Headache (CH) attacks during the time required for the preventative agent verapamil (V), to have effects [1]. However, both drugs are often unsatisfactory. We present the case of a woman, affected by Chronic Cluster Headache (CCH), successfully treated with high doses of C and V. Moreover, we treated similarly 20 Episodic Cluster Headache (ECH) patients with satisfactory results. A 62-years old housewife, in 2006 had an isolated cluster (ICHD-II) of 40 days duration, unresponsive to NSAIDs. In 2008, she had a second cluster, responsive to 6 mg sc sumatriptan; oxygen inhalation was ineffective. Patient was successfully treated with V 240 mg and prednisone (P) 50 mg/day per os for 7 days, tapered in a month.In January 2009, she had a new cluster, that became chronic with 2-8 attacks/24 hours, not responsive to P 50 mg/day, V 320 mg/day, lithium (750 mg/day), valproate (1000 mg/day). When we saw her, in August 2011, she had 5 attacks/24 h, despite taking V per os 320 mg/day. Patient was administered methylprednisolone (MP) 500 mg iv/day for 2 days, then 250 mg for 3 days, followed by P 25 mg per os for 2 days, tapered in 8 days. V was increased gradually to 600 mg/day. In the following month there were no attacks. In September, she presented 1-3 attacks/24h nocturnal and mild, lasting 15 min. V was increased to 680 mg/day with disappearance of attacks. In November, V was slowly reduced to 320 with no recurrence. To the best of our knowledge this is the first report about high doses of iv MP associated with high doses of V per os being effective in CCH. Data are being elaborated on a group of 20 ECH patients treated similarly with good results. If confirmed, our findings warrant the reevaluation of the doses and timing of these drugs in CH with appropriate clinical trials.
